# A single-item Expectancy Measure’s Validity, Reliability, and Responsiveness to Detect Changes in Clinical Efficacy Studies of Integrative Cancer Therapies: A Methodology Study

**DOI:** 10.1177/15347354241273944

**Published:** 2024-08-20

**Authors:** Anna Efverman

**Affiliations:** 1 University of Gävle, Sweden

**Keywords:** acupuncture, cancer care, category scale, contextual effects, numeric rating scale, nursing, oncology care, rehabilitation, treatment expectations, visual analog scale

## Abstract

**Background:**

Expectations may modify outcomes. However, studies often fail to measure expectations. This raises the need for a brief valid and reliable expectancy measure.

**Objectives:**

To study treatment expectations in individuals entering acupuncture or rest, validity and test re-test reliability of a single-item expectancy measure graded on a category scale, a Numeric Rating Scale (NRS) and a Visual Analog Scale (VAS), and to identify psychometric differences between the scales.

**Method:**

In this methodology study, treatment expectations were measured in 363 participants before they received acupuncture (genuine traditional penetrating or non-penetrating telescopic sham acupuncture, n = 239, 98%, responded) or a control treatment involving just rest (n = 120, 100%, responded), aimed to improve level of relaxation. A treatment expectancy measure, graded on a five-grade category scale, an eight-grade NRS and a 100 mm VAS, was tested for test re-test reliability. Level of expectation and relaxation was measured at baseline, pre- and post-therapy (n = 729 expectancy measurements).

**Results:**

The participants scheduled for acupuncture or rest believed moderately (Inter Quartile Range, IQR, moderately-much) and much (IQR moderately-much) the treatment to be effective. The Intra-Class Correlation coefficient versus Kappa coefficient between test and re-test was .868/.868 for the category scale, .820/.820 for the NRS, and .856/.854 for the VAS. The middle step “Believe moderately the treatment to be effective” was equivalent with median 4 (IQR, 3-4) on NRS and median 52 mm (IQR 42-52) on VAS. The response rates were 708 (97%) on the category scale, 707 (97%) on the NRS, and 703 (96%) on the VAS. All three scales discriminated that pre-therapy expectations were more positive in the individuals who reported an improvement in relaxation level (*P* < .001-.003). The VAS presented higher responsiveness to detect expectancy changes over time (71% increased expectation), compared to the NRS (52% increased) and the category scale (12% increased), *P* < .001.

**Conclusions:**

Individuals entering acupuncture, or a control intervention, presented positive treatment expectations, and the expectancy measure presented satisfactory reliability, validity, high response rates, sensitiveness, and responsiveness. Integrative cancer therapy researchers who want to control for expectancy-related bias in clinical trials should consider measuring expectation using the single-item expectancy measure.

## Introduction

Integrative oncology is a patient-centered, evidence-informed field of cancer care. Traditional, Complementary, and Integrative medicine (TCIM), for example acupuncture therapy, is utilized alongside conventional cancer care.^1^ Evidence-informed means that individualized treatment decisions are based on best available knowledge, for example knowledge on safety and effects of treatments derived from randomized, sham controlled intervention trials.^2^ Both TCIM intervention studies^3-5^ and conventional medicine intervention trials^6-9^ often lack treatment expectancy measurement. Expectations of recovery and treatment effects^10,11^ have been seen to highly modify treatment outcomes in both TCIM and in conventional medicine. Men and older patients expressed weaker beliefs in acupuncture effects than other patients undergoing cancer therapy, indicating the importance of collecting expectancy data in acupuncture studies to be able to treat expectancy as an effect-modifier.^1^ Integrative cancer therapy researchers would welcome an expectancy measure method, with satisfactory clinimetric properties,^12^ that induces low patient burden.

An expectation is a prediction based on reasoning and learning from experience. Predictions regarding the effect of treatments are often mentioned as treatment expectations.^10^ Most treatments may be divided into a specific treatment component, for example a pharmacological substance in a medication or skin-penetration during acupuncture, and a nonspecific component that includes the context surrounding the delivery of treatment.^13^ Effects of non-specific components of a treatment are often mentioned as placebo effects. Placebo effects are defined as positive treatment effects that cannot be attributed to active treatment components but are elicited by positive expectations or the psychosocial context in which treatment takes place.^13^ The pathways of placebo are often described as a complex interaction between psychological processes, for example, expectations, motivation, and hope to recover, social learned processes, for example, the pleasure of touch, and neurobiological mechanisms for example, endocrine, and immune functions.^14,15^ Expectancy is one of the key contributors of the placebo effect.^10,11,13^ Both the patient’s treatment expectations^1,10,11,16^ and the therapists’ enhancing of the patient’s treatment expectations^10,17^ have been seen to modify treatment effects. Of acupuncture-treated patients with low treatment expectancy, 60% experienced radiotherapy-induced frequent stools, compared to 26% of patients with more positive treatment expectations.^16^ In another randomized controlled acupuncture study, treatment expectancy did not modify the treatment outcome hot flushes.^18^ There are descriptions in the literature^19^ exemplifying that there exists a misunderstanding that placebo effects of significance only exist in TCIM methods, not in conventional medicine. Some believe that individuals entering TCIM methods have extraordinary positive treatment expectations, compared to individuals entering other treatments.^19^ Such attitudes may become barriers to integrating the methods within conventional approved care in cancer care.^2^ However, clinically significant placebo effects were observed during conventional medicine, for example during physical activity interventions,^8^ psychotherapy,^20^ pain- relieving injections^21^ and medications for asthma,^22^ depression,^12^ and migraine.^7^ During acupuncture, several studies found that the difference in treatment outcomes was even larger between patients with different levels of treatment expectations than the difference was between the compared randomization groups.^11,16,17^

In cancer care, expecting side effects of cancer therapies was associated with higher likelihood for experiencing these side effects in both therapy naïve and therapy-experienced patients.^23^ Cancer care researchers should accordingly assess participants’ treatment expectations and analyze the expectation as an outcome moderator. The participants’ expectancies can otherwise undermine the validity of the studies, since the risk for receiving placebo, sham, or other control treatments may lower positive treatment expectations.^24^ The impact of treatment expectations on psycho-neuro-immunological responses, which may affect treatment outcomes, was exemplified in a previous experimental study. During genuine acupuncture and credible sham acupuncture, inducing the same level of treatment expectation as genuine acupuncture, higher sensory activation in the brain was observed, compared to during a skin prick that was not expected to induce any therapeutic effect.^25^ Although expectations have been seen to modify treatment effects, most clinical acupuncture trials still do not measure expectations and analyze expectations as an outcome moderator. A diversity of expectancy measures has been used in acupuncture studies, almost all applied without any testing of validity and reliability.^3^ However, the credibility/expectancy questionnaire has been found to present satisfactory psychometric properties.^26^ The questionnaire includes 6 items regarding how successful the patient believes the treatment to be to reduce symptoms and how much improvement the patient thinks will occur. Accordingly, a fundamental criterion for using this questionnaire is that the patient experience symptoms when the treatment expectancy measure is conducted.^26^ In cancer care, acupuncture is often given to prevent symptoms from occurring, for example cancer therapy-induced nausea.^27^ The working alliance index partly targets treatment expectancy, for example by asking how confident the patient is in regarding the therapist’s ability to help him/her.^28^ This index may plausibly be used preceding preventive treatments. However, the original version includes 36 items^28^ and the short-version 12 items^29^; plausibly researchers consider multi-item psychometric expectancy-instruments to induce too much patient burden. According to the clinimetric tradition,^12^ researchers in TCIM^2^ may prefer single-item questions, developed according to the principal “one phenomenon—one question.”^13^ A clinimetric expectancy measure method should be valid, meaning that it measures the phenomenon supposed to be measured (treatment expectancy) in the target population. It should be reliable in terms of staying stable over time if no real changes occur, while it should have satisfactory responsiveness to detect changes over time if real changes occur.^12^ Responsiveness is defined as “the ability of a health-related patient-reported outcome instrument to detect change over time.”^30^ Further, when applying the expectancy grading as a treatment outcome moderator,^3^ the expectancy grading should be sensitive enough to discriminate between study participants who will differ in treatment outcomes.^12^ After recently systematically reviewing conducted acupuncture studies, researchers highlighted the need for a short expectancy grading, fulfilling the above-mentioned properties, to be used in future clinical trials.^11^

The objectives of this study were to compare treatment expectations between individuals entering acupuncture therapy or a control intervention, and to study validity and test re-test reliability of a treatment expectancy measure graded on a category scale, a Numeric Rating Scale (NRS) and a Visual Analog Scale (VAS). Further, the objective was to identify potential differences between the scales regarding response rates, responsiveness to detect changes in treatment expectations over time and to discriminate between participants with different treatment outcomes.

## Methods

This study was a methodology study, using expectancy data collected within a randomized controlled acupuncture cohort performed within an academic thesis (n = 363),^31^ compared to an ethically approved non-randomized reference cohort (ethical approval number: Umeå 2016/362-31, approval date 2017.01.10). The trial was, according to the Swedish ethical law (SFS 2003:460), ethically reviewed being included in the academic thesis at candidate level. The regional ethical committee approved the reference cohort and the comparison between the reference cohort and the acupuncture cohort. It complied with the declaration of Helsinki regarding ethical principles for medical research involving human subjects, and with the policies for TCIM research.^32^

### Recruitment

Participants (n = 363) were recruited by 12 therapists using convenience-sampling, either via personal communication or using a written flyer regarding the study. Both the personal initial contact and the flyer contained the same information, that is, “we are conducting a study regarding relaxing effects—would you like to receive further information?” When recruiting participants to the acupuncture cohort, the study information contained a message saying that the effect of acupuncture would be evaluated. When recruiting participants to the reference cohort, the study information contained the message saying that the effect of resting would be evaluated. The participants were individuals in general, not part of any specific patient population, mainly recruited from the therapists’ networks in their local community. They were screened for inclusion criteria: an age of 18 years or older, and physical, mental, and linguistic capacity to give informed consent, for example, understand Swedish. Exclusion criteria included previous education in acupuncture therapy, in terms of being an acupuncturist. All participants (the acupuncture cohort and the reference cohort, n = 363) were included when analyzing level of baseline treatment expectancy. In the methodological analysis, only individuals in the acupuncture cohort (n = 243, blindly scheduled for genuine or sham acupuncture) were included. All participants gave informed consent. The therapists made a time appointment for the “study day” with the individuals, a couple of days to a couple of weeks after the inclusion.

### Intervention

The reference cohort received no acupuncture, they just rested for 30 minutes as a control intervention. Immediately after the methodology testing and re-testing described below, the participants in the acupuncture cohort randomly (by use of statistician-delivered computerized randomization table) received one session of standardized genuine or sham (Park’s telescopic sham device) acupuncture. A written information letter informed the participants that they would be randomized to one of two types of acupuncture needles, and that the effect of either type was unknown. They were informed that one acupuncture type penetrated the skin and during the other acupuncture type, needles were placed against the skin—none of the acupuncture needling alternatives was cited as “sham” or “placebo.” The nine acupuncture treating physiotherapists (acupuncture experience ranged 2-20 years) bilaterally treated the traditional acupuncture point Pericardium 6 (PC6) in the genuine acupuncture group, since patients experienced relaxing effects during PC6-acupuncture in a previous study.^27^ In the sham acupuncture group, the physiotherapists bilaterally treated a sham point located four body-inches (one body-inch, or “cun” according to TCIM, is equivalent with the treated participant’s thumb) proximal and one body-inch radial from the PC6 point.^31^

### Data Collection

#### Socio-demographic and other baseline information

Two hours before acupuncture treatment or rest, the individuals delivered socio-demographic and other kinds of background information seen in Table 1. The reliable and valid Swedish version of the EuroQol Visual Analog Scale (EQ-VAS) measured self-perceived health status using a vertical scale anchored 100 (best imaginable health state) at the top and 0 (worst imaginable health state) at the bottom.^33^

**Table 1.** Characteristics of the Study Participants.

Variable	The total study group	The acupuncture cohort^a^	The reference cohort^b^
	n = 363	n = 243	n = 120
Sex, n (%)	n = 358	n = 238	
Man	99 (28)	58 (24)	41 (34)
Woman	259 (72)	180 (76)	79 (66)
Age in years, m, ±SD	n = 350	n = 230	45.7 ± 17.5
	43 + 15.0	41.8 ± 13.2	
Marital status, n (%)	n = 354	n = 234	
Married or living together	246 (69)	174 (74)	72 (60)
Partnered but live apart	32 (9)	24 (10)	8 (7)
No partner, that is, single	76 (31)	36 (16)	40 (33)
Education, n (%)	n = 358	n = 238	
Elementary school	6 (2)	4 (2)	3 (3)
Secondary	101 (28)	78 (33)	23 (19)
Graduate school or higher education	250 (69)	156 (65)	94 (78)
Self-perceived health status EQ-VAS, md, Interquartile range	n = 350	n = 230	80, 70-90
	80, 70-90	80, 70-90	
Had previously received acupuncture, n (%)	n = 344	n = 225	n = 119
Yes	160 (47)	108 (45)	52 (44)
No	197 (57)	130 (55)	67 (56)

Numbers (n) and proportions (%) of responding individuals are presented. N responding participants are given in case of missing data.

Abbreviations: md, median; m, mean; ±1 SD, standard deviation; EQ-VAS, EuroQoL-Visual Analog Scale: 0; worse imaginable health status to 100; best imaginable health state.

a The acupuncture cohort received genuine acupuncture or sham acupuncture.

b The reference cohort received no acupuncture, they just rested during the treatment session.

#### The single-item expectancy measure

The single-item expectancy measure covered the question: “Do you believe the treatment to be effective to provide effects?” The question regarded relaxing effects. In the acupuncture cohort and in the reference cohort, the words “treatment” and “effects” were thus clarified: “Do you believe the needling treatment to be effective to provide relaxing effects?,” and “Do you believe the rest to be effective to provide relaxing effects?,” respectively. The participants answered the single-item expectancy measure using three types of scales, graded in the same standardized order for all participants. The first scale was a five-category scale, with the answering alternatives “No I don’t believe the treatment to be effective” or “Yes, I believe a little/moderately/much/completely the treatment to be effective.” The second scale was an eighth grade (0-7) NRS with the anchors marked “I don’t believe at all the treatment to be effective” and “I completely believe the treatment to be effective.” The third scale was a 100 mm horizontal VAS, with the anchors marked like the NRS. All scales were answered using paper and pen. Accordingly, the participants marked their grading by drawing a vertical marking on the horizontal VAS (no ruler was used).

The single-item expectancy measure was developed and validated according to the clinimetric methodology,^12^ which is a is a highly established data collection method in cancer care. Clinimetrics concerns single-item rating scales and other expressions that are used to feasibly, validly, and reliably describe or measure distinctly clinical phenomena in line with how the target population describes the symptoms or phenomena.^12^ Accordingly, the single-item expectancy measure was formulated according to results from patient interviews. Then the category scale was tested for face validity during qualitative interviews with 9 patients, who described their interpretation of the expectancy question and the meaning of each answering alternative. Since acupuncture often is conducted preventively in cancer care,^27^ it was important that the formulation of the question allowed both patients experiencing and not experiencing symptoms to answer it. The single-item expectancy measure answered using the category scale was pilot tested in 10 patients answering it 3 times during 5 weeks of acupuncture therapy. Then, the category scale was used in a full-scale study (n = 215) regarding preventive and treating antiemetic effects of acupuncture.^27^

#### Measuring test re-test reliability and expectancy changes over time

To test re-test reliability, data was used from the participants in the acupuncture cohort only, regarding their responses to the single-item expectancy measure repeated twice. The first time was 2 hours before acupuncture treatment (test; baseline). At this time-point, the individuals answered the expectancy question in privacy (mostly in their home). The second time was just before treatment (re-test; pre-therapy). At this time-point, the individuals responded to the single-item expectancy measure in privacy at the same location as the location for the acupuncture treatment but without any attending therapist.

To be able to investigate changes in belief over time, the participants in the acupuncture cohort responded to the single-item expectancy measure again directly after the acupuncture treatment (post-therapy). At this time-point, the individuals answered the single-item expectancy measure in privacy at the same location as the location for the acupuncture treatment without any attending therapist.

#### Measuring differences in treatment outcomes

The study measured level of relaxation pre-therapy and post-therapy in the acupuncture cohort: “Do you feel relaxed right now?” graded on the established 100 mm VAS^34^ with the anchors marked “Not relaxed at all” and “Completely relaxed.” The participants marked their grading by drawing a vertical marking on the horizontal VAS using “paper and pen.” A change in level of relaxation of ≥10 mm was considered being clinically significant, based on previous studies.^34,35^

### Data Analyses

Descriptive statistics regarding median value and interquartile range of the 363 participants’ treatment expectation at baseline were presented, and the baseline expectancy gradings were compared between the acupuncture cohort and the reference cohort using Mann Whitney *U*-test. The methodological data analysis presents the total study acupuncture cohort (genuine and sham acupuncture treated participants) since the improvement in relaxation did not differ between the randomization groups (the groups improved median 26 and 30 mm, respectively).^31^ For the acupuncture cohort, Spearman’s correlation coefficients and Intra-Class Correlations (ICC) presented the co-variation between test and re-test regarding the participants’ answers on the category scale, NRS and VAS. Correlation coefficients lower than .25 were interpreted as “small or non-existent coefficients,” .26 to .49 as “low coefficients,” .50 to .69 as “moderate coefficients,” .70 to .89 as “high coefficients,” and .90 or higher as “very high co-efficients.”^36^ For analyzing agreement between test and re-test, a weighted kappa (quadratic weights) was used, interpreted as follows: 0.40 and below meant “weak agreement,” 0.41 to 0.60 “moderate agreement,” 0.61 to 0.80 “substantial agreement” and 0.81 to 1.00 “almost perfect agreement.^37^ For each step on the category scale, the participants’ variation (interquartile range) in NRS and VAS grading was calculated, being an observation of the scales’ validity to measure the same phenomenon,^38^ treatment expectations.

For the acupuncture cohort, answering rate in terms of number (n) and proportion (%) of participants who delivered data using the category scale, the NRS and the VAS was descriptively presented using a convergence table^39^ and statistically compared using Chi^2^ test. The single-item measure’s capacity to discriminate between participants with different treatment outcomes was calculated by comparing expectancy ratings with Mann Whitney *U*-test between participants who post-therapy (compared to pre-therapy) had improved ≥10 mm in graded level of relaxation and participants who had improved <10 mm in graded level of relaxation.^34,35^ The single-item measure’s responsiveness to detect changes in levels of expectancy when being responded to using the 3 scales was analyzed by comparing the proportion of participants who post-therapy compared to pre-therapy had changed at least 1 step on each of the expectancy-scales, using Chi^2^-test (improvement of at least one step on the VAS means an improvement of 10 mm). The analyses were performed in IBM Statistical Package of Social Science (SPSS) for Windows, version 24.0 (Armonk, NY: IBM Corp), except for Kappa coefficients calculated in MedCalc 12.6. The significance level was set at 5%.

## Results

### The Participants

**Figure 1.**
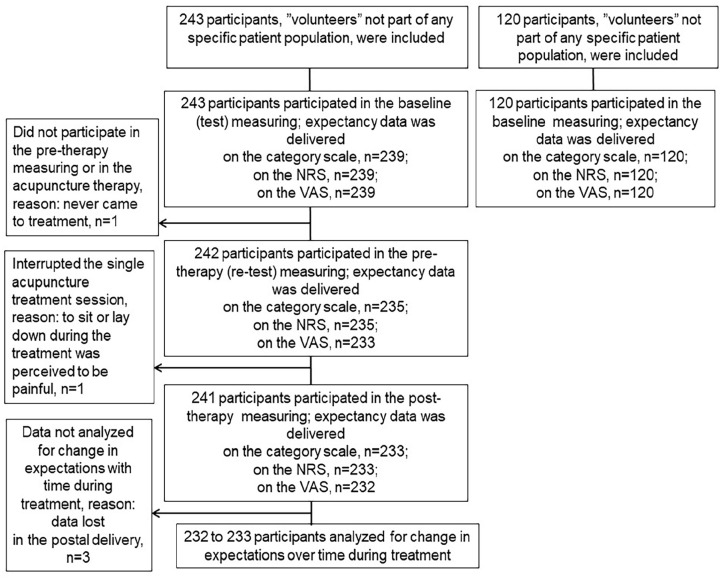
Flow-chart of the number of participants participating in the expectancy measurements. Data lost in postal delivery means the postal delivery from the cities of the data collection to the city of the evaluator.

The descriptive characteristics of the 363 study participants are presented in Table 1. Most participants were women (72% women, mean age 43), who graded their health status to be 80 (median value). The analysis of psychometric properties and responsiveness included the acupuncture cohort (n = 243). The number of participants who responded to the single-item expectancy measure at all three measurements (baseline, pre-treatment plus post-treatment) was 231 of 243 regarding the category expectancy scale, 231 regarding the NRS expectancy scale, and 230 regarding the VAS expectancy scale (Figure 1).

### The Participants’ Treatment Expectations Graded Using the Single-Item Expectancy Measure

The participants in the acupuncture cohort believed moderately (median value) in acupuncture to effectively give relaxing effects (IQR moderately-much) on the category scale, 4 (3-5) on the NRS, and 58 (42-72) on the VAS. The participants in the reference cohort believed much (median value) in rest to effectively give relaxing effects (IQR moderately-much) on the category scale, 5 (4-6) in the NRS, and 70 (50-80) on the VAS. The less positive expectancy in the acupuncture cohort than in the reference cohort were statistically significant (P < .001 for all scales).

### The Response Rates When the Single-Item Expectancy Measure Was Responded to Using Different Scales

Since the study applied the single-item expectancy measure 3 times in the 243 participants using the 3 scales, the total number of offered expectancy measurements using each scale was 729. Of the 729 offered measurements, the participants delivered answers to 708 (97%) measurements using the category scale, 707 (97%) using the NRS, and 703 (96%) using the VAS (P = .072). One single participant answered VAS without answering the category scale. No participants answered NRS in absence of answering the category scale (Table 2).

**Table 2.** Convergence Table of the Response Rates to the Single-Item Expectancy Measure Using a Category Scale, a Numeric Ratings Scale, and a Visual Analog Scale.

Variable	Answered category scale	Did not answer category scale	Total number of measurements
Answered NRS	707	0	707
Did not answer NRS	1	21	22
Total measurements category scale	708	21	729
Answered VAS	702	1	703
Did not answer VAS	6	20	26
Total measurements category scale	708	21	729

Number (n) is presented. The total n of offered single-item expectancy measurements on each type of scale was 729, since the 243 participants were asked to grade expectancy repeatedly, 3 times. NRS: Numeric Rating Scale. VAS: Visual Analog Scale.

### Test Re-test Reliability of the Single-Item Expectancy Measure

The correlations between the test and re-test single-item expectancy measure ranged from 0.823 to 0.871 for the different types of scales, which were interpreted as “high” coefficients.^36^ The agreement of 0.820 to 0.868 between test and re-test were interpreted to be “almost perfect”^37^ (Table 3).

**Table 3.** Co-variation and Agreement Between Test and Retest of the Single-Item Expectancy Measure.

Type of scale	Test	Retest	Spearman’s correlation coefficient, CI	Intra class correlation coefficient, CI	Weighted Kappa^a^ coefficient (standard error), CI
	md, (25th-75th percentile)	md, (25th-75th percentile)			
Category scale	n = 235	n = 233	n = 232	n = 232	n = 232
	moderate	moderate	0.871**	0.868**	0.868
	(moderate-much)	(moderate-much)	0.836-0.899	0.833-0897	(0.023)
					0823-0913
NRS	n = 235	n = 233	n = 232	n = 232	0.820
	4	4	0.823**	0.820	(0.036)
	(3-5)	(3-5)	0.776-0.860	0.773-0.858	0.749-0.890
VAS	n = 233	n = 232	n = 231	n = 231	0.854
	58	58	0.849**	0.856	(0.019)
	(42-72)	(39-72)	0.809-0.882	0.818-0.887	0.817 -0.891

Abbreviation: CI, 95% confidence interval.

a Using quadratic weights. The expectancy question: “Do you believe the needling treatment to be effective to provide (relaxing) effects?” Category scale: “No I don’t believe the treatment to be effective” or “Yes, I believe a little, moderate, much, or completely the treatment to be effective” (5 categories). NRS: Numeric Rating Scale (graded 0-7). VAS: Visual Analog Scale (graded 0-100 mm). The anchors of the NRS and the VAS were marked, “I don’t believe at all the treatment to be effective” and “I completely believe the treatment to be effective.”

** Correlation is significant at the .01 level (two-tailed).

### The Validity of the Single-Item Expectancy Measure

**Figure 2.**
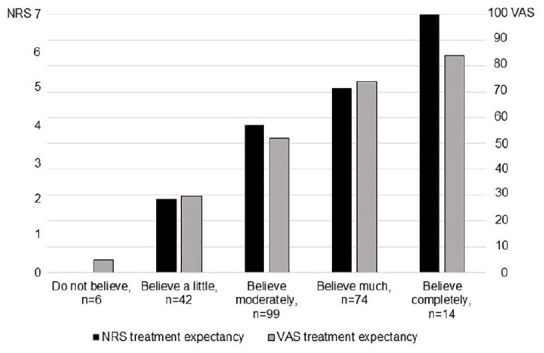
The median value on the expectancy NRS (Numeric Rating Scale) and the expectancy VAS (Visual Analog Scale) is presented, for each of the response alternatives on the expectancy category scale.

When analyzing the 235 delivered baseline single-item expectancy measures on NRS and VAS, compared to the single-item expectancy measures on the category scale, the NRS grading (median, interquartile range) for each category of the category scale, were; “Do not believe the treatment to be effective”: 0, 0 to 1.5, “Believe a little”: 2, 1.75 to 3, “Believe moderately”: 4, 3 to 4, “Believe much”: 5, 5 to 6, and “Believe completely”: 7, 6 to 7 (Figure 2). For each category of the expectancy category scale, the VAS (median, interquartile range) were; “Do not believe treatment to be effective”: 5, 1.5 to 19.5, “Believe a little”: 29.5, 20 to 37, “Believe moderately”: 52, 42 to 52, “Believe much”: 74, 68 to 81, and “Believe completely”: 84, 92 to 100 (Figure 2).

### The Single-Item Expectancy Measure’s Sensitivity to Discriminate Between Participants With Different Treatment Outcomes

**Figure 3.**
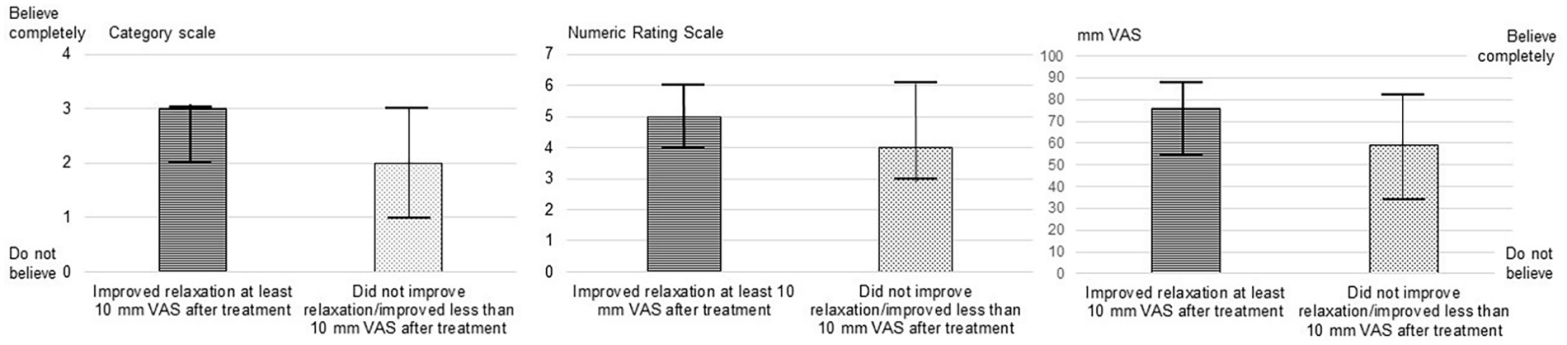
The median values and interquartile ranges on the pre-therapy expectancy grading on the category scale, the NRS (Numeric Rating Scale) and the VAS (Visual Analog Scale) are presented in the participants who improved level of relaxation (an improvement of at least 10 mm between pre- and post-therapy) and in participants who did not improve level of relaxation (improved less than 10 mm).

The pre-therapy expectancy grading on the category scale (*P* < .001), the NRS (*P* = .003), and the VAS (*P* = .002) discriminated that the pre-therapy expectations were more positive in the participants who reported an improvement in relaxation level of at least 10 mm (pre- to post-therapy) compared to the participants who did not improve (Figure 3).

Table 4 presents the probability to improve at least 10 mm in the treatment outcome level of relaxation (pre- to post-therapy) in participants who increased level of treatment expectancy at least 1 step (pre- to post-therapy) compared to participants who did not increase level of expectancy. The probability to improve in relaxation was higher in participants who increased treatment expectancy graded on the NRS (81% improved in level of relaxation) compared to participants who did not increase expectancy (65% improved in level of relaxation). The probability to improve in relaxation was also higher in participants who increased treatment expectancy graded on the VAS (77% improved in level of relaxation) compared to participants who did not increase expectancy (63% improved in level of relaxation) (Table 4).

**Table 4.** The Single-Item Expectancy Measure’s Capacity to Discriminate Between Participants who Reported an Improvement in Relaxation Level After Treatment and Participants who did not Improve in Relaxation Level.

Variable	Improved relaxation ≥10 mm post-therapy^a^	Did not improve relaxation or improved <10 mm post-therapy^a^	Relative risk for improvement, CI	P-value comparison
Total numbers	168	62		
Category scale				.837
Increased in category scale measured treatment expectation	20 (71)	8 (29)	0.92, 0.43-1.99	
Did not increase category scale measured treatment expectation	148 (73)	54 (27)	Ref.	
NRS				.007*
Increased in NRS measured treatment expectation	96 (81)	23 (19)	1.54, 1.09-2.19*	
Did not increase in NRS measured treatment expectation	72 (65)	39 (35)	Ref.	
VAS				.027*
Increased in VAS measured treatment expectation	127 (77)	37 (23)	1.25, 1.01-1.55*	
Did not increase in VAS measured treatment expectation	41 (63)	24 (37)	Ref.	

Abbreviation: CI, 95% confidence interval.

a Compared to pre-therapy.

* Statistically significant difference in relaxation outcome between participants who increased treatment expectancy and participants who did not increase level of treatment expectancy. Category scale: “No I don’t believe the treatment to be effective” or “Yes, I believe a little, moderate, much, or completely the treatment to be effective” (5-graded). NRS: Numeric Rating Scale (graded 0-7). VAS: Visual Analog Scale (graded 0-100 mm). The anchors of the NRS and the VAS were marked; “I don’t believe at all the treatment to be effective” and “I completely believe the treatment to be effective.”

### The Responsiveness of the Single-Item Measure to Detect Changes in Expectancy Over Time

**Figure 4.**
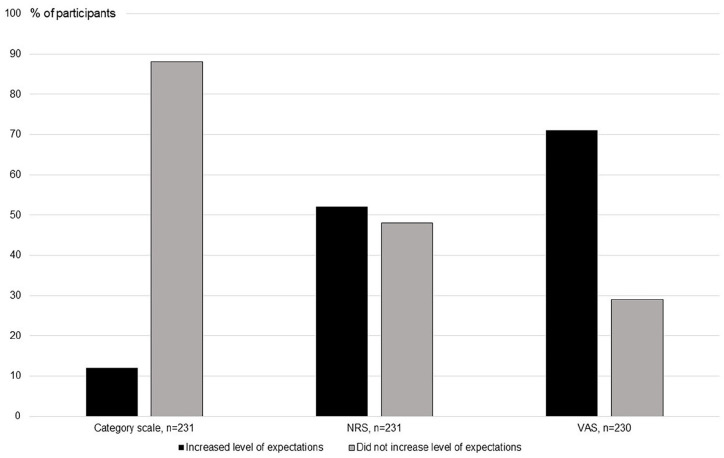
Proportions of participants who were improved, or not improved, regarding level of expectations over time between pre- and post-therapy, when grading expectancy on the category scale, the NRS (Numeric Rating Scale) and the VAS (Visual Analog Scale). Improved means grading more positive expectations. n, number, of respondents is given.

The VAS presented higher responsiveness to detect changes in treatment expectancy over time, pre- to post-therapy (n = 164, 71% increased expectation), compared to the NRS (n = 119, 52% increased expectations, *P* < .001) and to the category scale (n = 28, 12% increased expectations, *P* < .001) (Figure 4).

## Discussion

This study found that both individuals entering acupuncture or a control intervention presented positive treatment expectations, and the single-item expectancy measure method presented satisfactory reliability, validity, high response rates, sensitiveness, and responsiveness. The single-item expectancy measure was reliable, and the response rates were high, irrespective of whether expectancy was graded using a category scale, NRS or VAS. The NRS and the VAS were valid in terms of agreement to the previously used^27^ category scale. The category scale, NRS and VAS were sensitive enough to discriminate that pre-therapy expectations were more positive in participants who reported an improvement in relaxation level than in participants who did not improve. The expectancy VAS presented the highest responsiveness to detect changes in expectations over time.

Three-armed acupuncture studies randomizing patients to acupuncture, sham acupuncture, or a control group receiving no acupuncture risk less positive treatment expectancy in the control group, since the patients may be disappointed by the lack of acupuncture treatment. Their negative expectations may then plausibly affect their outcomes, causing the researchers to over-estimate the effects of the sham acupuncture treatment compared to the effects of no acupuncture at all. In the current study, the control group receiving no acupuncture, just rest, was a non-randomized reference cohort recruited separately. The acupuncture cohort was compared with a reference group, not to a third randomized arm. That design may have the benefit of avoiding the impact of expectancy per se on the results. When conducting a three-armed randomized controlled trial, potential disappointment on being allocated to the non-treated group is likely to decrease positive treatment expectations immediately when the participants become aware of the randomization result. The finding that positive treatment expectations occurred both in individuals scheduled for acupuncture or a control treatment covering no acupuncture at all supports the choice of designing two-armed randomized controlled studies with a non-randomized control group in some preferable situations.^23,24^ Since the individuals entering acupuncture did not grade more positive treatment expectations than individuals entering the control treatment involving just rest, this supports the falsification of statements that placebo effects of significance only exist in TCIM methods, not in conventional medicine.^19^

Satisfactorily, the reliability of the single-item expectancy measure was high. The reliability of most other expectancy measure methods used in acupuncture studies have been poorly evaluated.^3^ One example of a previous study testing re-test reliability, used the expectancy question: “How confident do you feel that this treatment can alleviate your complaint” (6-point Likert scale). However, the reliability testing was biased by ongoing treatment between test and re-test.^40^ Reliability is typically defined as the ratio of variability between scores of the same study participants, at different times. High correlation coefficient means that there is little residual variability to confound good discriminations among participants’ scores.^37,38^ This occurred in the current study because the range of obtained scores from one and the same participant during test and re-test was low. The expectancy measuring scales accordingly produced stable measures over time during the period between baseline and pre-therapy, preceding the acupuncture treatment.

The valid previously used^27^ single-item expectancy measure responded to using the category scale divided the NRS and the VAS into almost equally sized parts that did not overlap each other, indicating the measure’s validity for measuring different levels of treatment expectations. A review of expectancy measure methods in acupuncture studies^3^ did not find any validity tested expectancy measure method, besides that face validity and internal consistency had been investigated for expected acupuncture effects for psychiatric symptoms (5-point Likert scale).^41^ However, data sets generated with Likert-type scales often have a skewed or polarized distribution, for example most study participants strongly agreeing or strongly disagreeing.^42^ Barth et al^43^ clearly described the formulation of their expectancy question, answered on a four-category scale (total n = 555). However, the authors did not mention its methodological properties. In line with the observations of the current study, the category scale, the NRS and the VAS presented high agreement when used for measuring pain.^34,44^

In the current study, the response rates were high for the single-item expectancy measure using the category scale, the NRS and the VAS. When applying the category scale, the NRS and the VAS for measuring pain, the studies presented lower response rates for VAS compared to the other types of scales.^3,45^ However, previous studies often covered older populations than the participants of this study. The study participants in the current study were rather young, mean age 42 years, and 65% had a higher education. They were plausibly highly motivated to respond to the questions since they were going to receive acupuncture treatment or rest treatment for relaxing purposes after the baseline and the pre-therapy single-item expectancy measure, and had just received the acupuncture treatment when answering the post-therapy measuring. The current study did not ask the participants which type of scale they preferred. Previous studies have shown that the less educated and the elderly preferred the category scale.^44,45^ Most incorrect responses, made by mistake, were seen to be delivered using the VAS.^45^ In the current study, the rather young and well-educated participants delivered just a couple fewer (ie, not statistically significant fewer) answers on the expectancy VAS (n = 703) than on the NRS (n = 707) and on the category scale (n = 708).

Besides stability during periods when no real changes occur, it is as important that expectancy measure methods can discriminate between groups of participants who differ from each other. For example, men and older patients expressed weaker beliefs in acupuncture effects than other patients undergoing cancer therapy.^1^ The single-item expectancy measure responded to using the category scale, NRS and VAS was sensitive enough to discriminate between individuals with different treatment outcomes; individuals who improved in relaxation had more positive pre-therapy expectations. Participants who did not increase level of expectation according to the category scale, however, did not differ in the treatment outcome relaxation, compared to the participants who did increase level of expectation. This may be explained by the fact that a “ceiling effect” probably occurred; most participants (82%) believed at least moderately that the treatment would be effective at the pre-therapy expectancy measure. According to the expectancy VAS, the participants who increased just 1 single step (1 mm) on expectancy VAS between pre- and post-therapy measuring had 22% higher probability to improve in level of relaxation by at least 10 mm on relaxation VAS. This observation indicates that even a small change in expectancy over time may be considered clinically significant in future clinical studies. The single-item expectancy measure method was able to discriminate between participants with different treatment outcomes, irrespective of the type of scale for grading expectancy. This underscores the relevance of including an expectancy measure in clinical intervention studies,^3,10,11,16,24^ since unmeasured treatment expectation may threaten the internal validity of studies.^3,24,46^ Particularly highly positive or highly negative treatment expectancies might make the detection of between-group differences more difficult. Expectancy multiplied with the treatment effects could result in false findings where unmeasured expectancy occur unequally between randomization groups.^24,47^ Indeed demonstration of positive treatment outcomes might be restricted by lack of or inadequate measurements.^46^ Measuring treatment expectations and thus including treatment expectation as a factor modifying the treatment outcome in the statistical analysis may reduce the impact of such bias.^3,43^

The finding that the expectancy VAS presented the highest responsiveness to detect changes in expectations compared to the NRS and the category scale, was an expected finding.^34^ There are 101 steps in a 100 mm VAS; the high number of steps make it easy to change in grading, resulting in ease of detecting statistically significantly changes also in small study populations.^48^ In a previous acupuncture study, the less sensitive expectancy category scale was used. The study failed to detect changes over time since almost all the patients graded pre-therapy that they believed moderately or much that the treatment was effective.^27^ In line with this, Barth et al^43^ also suggested that expectancy measurements with high responsiveness should be developed to capture the entire range of expectations in a more gradual way.

In this study, there several methodological choices were made. Although this paper used data from a randomized sham-controlled trial with a non-randomized reference group,^31^ the current paper compared expectancy data between the reference cohort and the total acupuncture cohort (genuine *or* sham acupuncture) since the improvement in relaxation did not differ between the randomization groups.^31^ The participants answered the single-item expectancy measure using 3 common types of scales. The standardized order placed the simple category scale first and the more complicated VAS last in the questionnaire, based on findings on response rates in previous studies.^34,45^ Data were analyzed using methods appropriate to the ordinal and category nature of the data.^39,42^ VAS data was considered to be ordinal data, in line with previous literature^39,42^ although other studies sometimes analyze VAS as if had been a continues scale.^34^ Weighted kappa was used, since an un-weighted kappa is based only on the proportion of exactly accurately repeated responses during re-testing compared to testing. This is not applicable for a 100 mm VAS. Since symptom-grading on VAS has not been seen to be proportional to stimulus-intensity within VAS’ entire length,^39^ a linear kappa did not seem accurate. For investigating if the expectancy NRS and the expectancy VAS were valid in terms of measuring the same phenomenon compared to the previously validated^27^ expectancy category scale, the study described agreement between the category scale and the NRS and the VAS, presenting the median and the interquartile range. These kinds of simple descriptive presentations have been suggested to be suitable measures of agreement because they provide this kind of information in a simple manner.^38^ Strengths of the study are the rather high number of participants, n = 363, and the sufficient response rates when the category scale, the NRS, or the VAS was used for grading expectations. It seemed highly important to study an expectancy measure associated with low patient burden, making it possible for researchers to apply it repeatedly during a trial instead of just measuring baseline expectancy,^48^ since patients’ expectations may change over time, affected by for example, information from therapists.^49,50^

A study limitation is that the treatment was very short, compared to an entire treatment period of for example, ten acupuncture treatments. Further, the follow-up period was short as well. There were only 2 hours between test and re-test, which is a short period when it comes to test re-test reliability testing. However, an expectancy grading based on hope for improvement may change a lot over time depending on the significance of emotions for patients’ statements,^50^ which might have induced bias in the re-test reliability testing if the study had adopted a longer period between test and re-test. The convenience sampling resulted in skewed gender distribution, rather young participants, and good health status compared to various cancer populations, which may limit the generalizability of the findings to patient populations with severe conditions. The single-item expectancy measure was adopted in individuals entering acupuncture or rest. Further validation of this single-item expectancy measure when used during other therapies than acupuncture is welcomed, for both conventional and TCIM methods, for broader dissemination and implementation. However, the single-item expectancy measure method has previously been used in rather frail patients undergoing cancer therapy.^16,27^

Patients treated with conventional antiemetics, and patients treated with acupuncture during emetogenic chemotherapy, expressed that their experiences and expectations were highly important to shape new experiences during their burdensome chemotherapy.^51^ In the light of the importance of expectations and that the fact that efficacy studies of integrative cancer therapies often do not apply any treatment expectancy measure method, and thus miss the chance to analyze treatment expectancy as a confounding factor or as an effect moderator, this study brings important implications. To summarize, individuals entering acupuncture, or a control intervention, presented positive treatment expectations, and the single-item expectancy measure method was reliable and valid, produced high response rates and was able to discriminate between individuals with different treatment outcomes, irrespective of whether it was graded on a category scale, a NRS or a VAS. The VAS presented the highest responsiveness scale to detect changes in treatment expectancy over time. Considering the satisfactory psychometric properties and low burden on the respondents, researchers who want to control for expectancy-related bias in clinical trials of integrative cancer therapies should consider measuring expectation using the single-item expectancy measure, graded using a category scale, NRS, or VAS. If researchers prioritize a scale that is easy to understand, the category scale may be preferable. If researchers prioritize a sensitive scale to detect changes over time, the expectancy VAS grading may be preferable to the category scale and the NRS.
